# How digital leadership adds affective commitment of new generation employees: an affective events perspective

**DOI:** 10.3389/fpsyg.2024.1476047

**Published:** 2025-01-07

**Authors:** Hongting Li, Futian Li, Junyi Ma, Bo Liu

**Affiliations:** ^1^School of Economics and Management, Southwest University of Science and Technology, Mianyang, Sichuan, China; ^2^Shanghai State Owned Assets and State Owned Enterprise Reform and Development Research Center, Shanghai, China

**Keywords:** digital leadership, new generation employees, affective commitment, employee voice behavior, employee empowerment

## Abstract

How do leaders’ responses to the digital era affect new generation employees’ affective commitment? As digital leaders have led to new ways of distributing digital resources and building virtual relationships, employees are facing a shift in the way of interaction, which influences their affective response to organizations. This study aims to understand how digital leaders interact with new generation employees to influence changes in employees’ affective commitment to organizations. We have developed a chain mediating model and tested it on data collected from 408 new generation employees working in China. Ultimately, we found that digital leadership is associated with more positive changes in new generation employees’ affective commitment. Furthermore, both employee empowerment and employee voice behavior mediate the relationship between digital leadership and affective commitment, forming a chain mediation mechanism in this relationship. We conclude with a discussion of theoretical implications and practical applications.

## 1 Introduction

With the steady increase in the proportion of new generation employees in the Chinese workforce, organizational leaders are increasingly encountering challenges related to demographics, including strained workplace relationships and frequent turnover (Lu et al., 2023). These issues not only disrupt short-term operational efficiency but also erode the long-term competitiveness of enterprises. Affective commitment, defined as the emotional attachment and identification of employees with their organization (Allen et al., 2017), plays a pivotal role in determining their decision to remain with or leave an organization (Chordiya et al., 2017). Lower levels of affective commitment among new generation employees have been strongly associated with higher turnover rates (Fazio et al., 2017). Leadership has always been a key factor in influencing employees’ affective commitment (Grego-Planer, 2022). As such, understanding leadership style is vital for building a talent pool and ensuring the sustained prosperity and innovation of enterprises.

Scholars have extensively examined generational differences in workplace attitudes and behaviors, identifying distinct variations across generations (Jones et al., 2018; Stewart et al., 2017). According to generational cohort theory, individuals belonging to different generational cohorts exhibit unique personality traits, motivational drivers, and behavioral patterns in the workplace, shaped by shared formative experiences during their preadult years, such as significant socio-economic events, natural disasters, or technological advancements (Okros, 2020; Cvetković et al., 2024). Since the 1990s, China has undergone systemic transformations in its economic, social, and cultural domains. The combined effects of the one-child policy, economic prosperity, internet proliferation, the expansion of higher education, and globalization have created a distinct growth environment and behavioral patterns for the new generation employees, markedly different from those of the preceding generation (Zeng and Greenfield, 2015; Garikapati et al., 2016). First, as digital natives, the new generation employees are immersed in vast amounts of information and have developed a strong preference for online communication, instant feedback, and looking for solutions on the Internet (Myers and Sadaghiani, 2010). Besides, influenced by globalization, they often embrace diverse values, maintain an open mindset, and advocate for freedom and autonomy (Angeline, 2011). Second, many new generation employees who are only children with superior material living condition exhibit traits such as individualism, confidence, and adaptability to change (Twenge and Campbell, 2012). Meanwhile, they also express a pronounced need for support, respect, and recognition from their leaders (Mencl and Lester, 2014). The meaning of work and self-transcendence is also what they are committed to pursuing (Kismono, 2023). Third, new generation employees possess higher education backgrounds but experience significant peer pressure, which fosters a strong sense of competitiveness and a drive for continuous self-improvement (Zhang et al., 2024). Thus, they seek to challenging tasks and opportunities to realize their self-worth at work. Consequently, the affective commitment of new generation employees is typically derived from the fulfillment of their needs for meaning, respect and self-development, rather than purely compensation or rank (Atrizka et al., 2020). This generational shift has led to higher expectations for leadership capabilities compared to older employees. Great leaders can recognize and adapt to these differences, and inspire the affective commitment of new generation employees.

Leadership is regard as a key factor in fostering the affective commitment of new generation employees (Semedo et al., 2016). Among them, responsible leadership, transformational leadership and ethical leadership have garnered significant attention (Haque et al., 2019; Ribeiro et al., 2018; Asif et al., 2019). Unlike these leadership styles that focus on result-oriented tasks, driving change and maintaining ethical standards, digital leadership is uniquely equipped to address the specific needs of new generation employees (Hazni and Nurhaida, 2024). By digital tools, digital leaders can facilitate online collaboration, provide immediate feedback, offer personalized career advancement and empower employees (Hazni and Nurhaida, 2024). For new generation employees, who value openness, autonomy and self-realization, digital leadership may be arguably a more suitable fit, as it effectively promotes their affective commitment to organization (Zeike et al., 2019). However, the impact of digital leadership in virtual contexts, as well as the psychological traits of new generation employees, have received little attention from scholars, which weakens the integrity and persuasiveness of the research. Besides, while prior research has noted the role of digital leadership in promoting the organizational citizenship behavior, the core mediating role of affective commitment has been neglected (Benitez et al., 2022). Therefore, current research has difficulty in explaining the interaction between digital leaders and new generation employees, as well as the antecedents of affective commitment of new generation employees. According to affective events theory, employees’ emotions fluctuate in response to interactions with their leaders (Weiss and Cropanzano, 1996). Digital leaders who are adept at using technology to identify and respond to the individual needs of new generation employees, can influence these affective events. This, in turn, has a direct impact on their affective commitment.

Affective events theory posits that employees’ affections are shaped by various events they experience in the workplace (Cropanzano et al., 2017). In the digital era, where organizational structure is flattened, leaders’ authority is decentralized and collaboration online prevails, digital leaders are more inclined to delegate the authority and foster employees’ competencies (Ansong and Boateng, 2019; Yang et al., 2024). Given that the needs of new generation employees for autonomy and self-realization are built on a certain amount of power, empowerment of digital leaders can generate positive emotional responses as a positive event (Ho et al., 2017). It follows from this that employee empowerment plays a critical role as a bridge between digital leadership and affective commitment of new generation employees. It emphasizes the importance of fostering emotional connections between leaders and new generation employees.

Moreover, the emotional relationships between new generation employees and organizations needs to be built through active participation and expression of employees (MacGeorge et al., 2017). According to affective events theory, the intensity of affective responses is directly related to the level of involvement in workplace events (Zhang, 2013). Voice behavior, initiated by employees, represents a deeply engaged event that can provoke strong emotional reactions. Specifically, with access to autonomy and digital resources—such as information, knowledge, and media platforms— new generation employees are more inclined to initiate interactive events with digital leaders, including voice behavior (Wan and Duffy, 2022). Regardless of whether the received feedback is positive or negative, voice behavior is perceived as a challenging task and a major event (Kim et al., 2016). Especially for new generation employees, who prioritize showcasing and enhancing their capabilities, these interactions are often viewed as positive events, further reinforcing their affective commitment (Zhu et al., 2015). Thus, employee voice behavior should be integrated into the framework linking digital leadership and affective commitment. Given that new generation employees with a high perception of empowerment are prone to voice their opinions boldly, employee empowerment and voice behavior serve as chain mediating roles in the relationship between digital leadership and affective commitment.

Simultaneously, from the social learning literature, existing studies emphasize the critical role of leadership styles such as responsible leadership and ethical leadership, arguing that the exemplary role of leaders significantly affects employees’ cognition and emotions (Haque et al., 2019; Asif et al., 2019). However, individual emotions are deeply rooted in the interactive events with leaders. Employees are more likely to generate affective commitment when they perceive interactive events as positive events (Panaccio and Vandenberghe, 2012). Despite this, discussions on how the employees, especially new generation employees, evaluate interactive events and specific measures leaders can take affectively interact with employees remain inadequate. Additionally, while existing researches highlight the impacts of affective commitment on voice behavior, it often overlooks the unique psychological needs of new generation employees (Wang et al., 2014). This cohort typically has heightened expectations for leadership and is less likely to establish affective commitment at the outset (Vandenberghe et al., 2004). As such, they are more inclined to follow a path characterized by “interactive events leading to affections” rather than the traditional sequence of “affections leading to events.”

In summary, consistent with affective events theory, this study considers (1) How digital leadership, as the initiating event, influences the affective commitment of new generation employees, (2) What roles employee empowerment and voice behavior play. Through the empirically analyzed 408 questionnaire data, we reveal that (1) Digital leadership has a significant positive impact on the affective commitment of new generation employees. (2) Employee empowerment and voice behavior partially mediate the relationship between digital leadership and affective commitment. (3) Employee empowerment and voice behavior further act as chain mediators in linking digital leadership and affective commitment. The research not only widens the understanding of affective connections between digital leaders and new generation employees, but also provides insights into the mechanism underlying the formation of affective commitment.

This article is organized as follows. Section 2 provides the literature review and hypotheses. Section 3 covers the method of investigation and analysis. Section 4 shows the results. Section 5 covers theoretical and practical implications, limitations and directions for future research.

## 2 Literature review and hypotheses

### 2.1 Digital leadership

Digital leadership refers to the process by which leaders use digital technology to promote changes in attitudes, emotions, and behaviors of employees, teams and organizations, thereby facilitating the transformation of enterprises (Kapucu, 2020). Such leaders are often distinguished by online communication, digital literacy, talent cultivation and a calling for digital transformation (Sainger, 2018). In practice, digital leaders can deliver personalized feedback and learning resources through a variety of digital platforms, such as internal social media, online training systems, data-driven performance management and more (Petrucci and Rivera, 2018). Besides, big data analytics enables digital leaders to analyze employees’ skills, interests, and potential, thereby providing training resources and promotion pathways for employees (Zia et al., 2024). Additionally, online communication tools such as virtual meetings and instant messaging are also central to digital leadership. They also tend to create flexible digital platforms where employees are encouraged to share ideas and solutions and take ownership of specific tasks (Antonopoulou et al., 2021). While most scholars stress the role of digital leadership in advancing digital transformation, the development of digital leader-member relationships within digital contexts remains underexplored. These interactive relationships, driven by digital leaders, significantly affect employees’ emotional responses. From the perspective of affective events theory, digital leadership acts as a driving force behind work events and serves as a key determinant of employee emotions, which is explored in this study.

### 2.2 Digital leadership and affective commitment of new generation employees

Affective commitment, as a positive affective response, represents the degree of employees’ affective connections to and identification with the organization (Allen et al., 2017). The form of affective commitment is linked to several desirable work outcomes, including career continuity, a sense of identification and honor for organizational membership (Swalhi et al., 2017). According to the affective events theory, personality traits affect how individuals cognitively assess events, which in turn shapes their emotional responses (Allen et al., 2017). Characterized by traits such as work autonomy, individualism and an open mindset, new generation employees prefer the leaders who provide personalized support, empowerment, flexible work arrangements and instant feedback. Differentiated with traditional leaders who often rely on absolute authority, imperative communications, and results-oriented tasks, digital leadership is better equipped to meet the needs of new generation employees for growth, openness and fairness, autonomy, self-actualization and emotional support at work, which helps to improve their affective commitment (Antonopoulou et al., 2021).

Specially, digital leaders are adept at creating efficient and transparent work environments by digital tools and resources (e.g., performance assessment tools, and personalized training systems). Their support for employees’ professional growth is perceived by new generation employees, which increases their sense of competence and control over their work. Second, digital leaders are open to innovative ideas of new generation employees and foster a climate of psychological safety by anonymous feedback channels and online collaboration tools (Erhan et al., 2022). It enables employees to express their ideas freely without fear of negative consequences, thereby cultivating a sense of participation and belonging. Third, digital leaders use digital tools to analyze the data on the specific needs of new generation employees and develop personalized ways of communications and incentives. It makes employees feel valued and supported, enhancing the emotional connection between employees and leaders. Finally, digital leaders empower new generation employees by granting decision-making authority and providing transparent information channels through the digital management platform, so as to enhance their sense of autonomy and trust in the organization. Besides, in the process of digital transformation, digital leaders also demonstrate the ability to cope with the challenges of internal or external environments and seize digital opportunities for organizational development. Their digital proficiency and visionary leadership attract new generation employees to follow, enhance a sense of optimism about the organizational future, and strengthen their loyalty. To sum up, we hypothesize the following:

*H1*: Digital leadership is positively associated with the affective commitment of new generation employees.

### 2.3 The mediation role of employee empowerment

Given that employee empowerment is a positive psychological perception of employees, it is likely to create the discernment that they can autonomously plan work processes and influence work outcomes based on confidence in their abilities and knowledge (Hirzel et al., 2017). Thus, when employees possess the necessary skills and resources to cope with their jobs, they are more likely to take ownership of work. Leaders can achieve employee empowerment in a variety of formal and informal ways, including, but not limited to, providing resources and information, encouraging participation in management, granting autonomy, providing feedback, and other methods (Yang et al., 2024). New generation employees with perceptions of empowerment are more motivated to engage in work and extra-role behaviors, which positively predict their career stability (Islam and Tariq, 2018).

In a dynamic adaptive atmosphere, digital leaders value employees’ continuous learning and professional development and promote the trend of decentralization of digital work. They are able to empower employees to take ownership of work tasks and emphasize the development of digital skills through digital technology (Eberl and Drews, 2021). It promotes employees’ understanding of their work goals and enhances their sense of job control and influence. Secondly, by optimizing workflow, resource allocation, and communication methods, digital leadership breaks down resource barriers, builds sharing channels, and enables the flow of all kinds of resources, materials and information in the social network. In this way, it helps the new generation employees to access, transfer and integrate high-quality resources and improves the perception of employee empowerment (Zeike et al., 2019). Finally, leaders are able to use digital technology to realize digital talent management. Digital tools are used to transmit organizational development strategies to employees and to give meaning to their jobs. Employees can then construct meaning for their work and develop the motivation and behavior to improve the organization’s status quo (Türk, 2023).

Further, employees with a high level of empowerment perception have their social and belonging needs met after feeling valued and recognized by the organization (Suarez-Balcazar, 2020). They thus develop emotional attachment and identification with the organization. Therefore, employee empowerment can positively predict an individual’s level of affective commitment. Based on this, we hypothesize the following:

*H2*: Employee Empowerment plays a mediation role in the relationship between digital leadership and the affective commitment of new generation employees.

### 2.4 The mediation role of employee voice behavior

Employee voice behavior refers to a spontaneous behavior in which employees put forward constructive opinions for the purpose of improving performance or solving problems (Liang et al., 2012). It is usually manifested in providing ideas for improvement in the decision-making, process, system or other optimizations, either within or outside the scope of their duties. Employee voice behavior has certain characteristics such as initiative, risk and constructive. From the affective events theory, the interaction between leaders and new generation employees can be regarded as a process of emotional exchange (Nazir et al., 2018). Compared with leader-initiated events, employee-initiated events have a stronger impact on the emotional responses of employees.

Digital leadership specializes in building knowledge resource systems and online learning platforms through digital technology (Benitez et al., 2022). This significantly helps employees gain more knowledge and insight, enhancing their self-efficacy to express their opinions. As for new generation employees, they are more likely to perceive a high level of control over the voice risk and motivated to perform it (Liang et al., 2012). Second, digital leaders are open to employees’ suggestions and promote a digitally-enabled voice climate where employees freely express their opinions. Consequently, a strong psychological safety can ease the felt risk of presenting new ideas and lead to a more positive evaluation of voice. Finally, digital leaders are able to provide personalized feedback and incentive on advice by digital tools (de Araujo et al., 2021). High-quality interactions strengthen employees’ positive attitudinal evaluation of voice and thus are more likely to engage in voice.

Affective commitment is an enduring psychological state that stems from feeling comfortable and competent in one’s job and workplace (Allen and Meyer, 1990). By expressing opinions, employees may feel fulfillment of their psychological needs and thereby increase the emotional investment to organizations (Kim and Leach, 2020). First, affective events theory proposes that individuals’ emotions fluctuate in response to interactive events with others (Weiss and Cropanzano, 1996). By exercising voice, employees may perceive the potential to influence decisions, and have the positive feelings of personal validation and self-worth (Frese et al., 1999; Jena et al., 2017). Especially, for new generation employees, they are committed to pursuing the meaning of work and realizing self-worth (Zhou and Qian, 2021; Westerman and Yamamura, 2007). When opinions are heard and acted upon, new generation employees may feel more comfortable knowing that their competence is acknowledged and contributes to the functioning of organization (Truss et al., 2012; Yoon, 2012). Besides, Voice behavior strengthens psychological empowerment of new generation employees, when they can influence the positive change processes of organization that are deemed necessary (Morin et al., 2016; Kim and Leach, 2020). They are more likely to perceive the respective demands for self-worth and self-realization met and thereby experience higher level of affective commitment (Zhang et al., 2024; Greguras and Diefendorff, 2009). Second, according to affective events theory, the intensity of affective responses is directly related to the level of engagement in workplace events (Zhang, 2013). Voice behavior puts new generation employees into organizational decisions making and emphasis on employee autonomy, particularly in times of major change (Heffernan and Dundon, 2015; Wu and Zhou, 2020). It helps to foster a sense of ownership and deeply involve in management, which assist employees in accepting, believing in, and identifying with organizational goals, thereby resulting in increased levels of affective commitment (Farndale et al., 2011). Third, the two-way and fair communications with digital leaders engender the belief of new generation employees that their contributions are valued and recognized (Whiting et al., 2012). If new generation employees have more needs for respect met, they will be more emotionally motivated (Korzynski, 2013). These positive interactions may direct to establish their psychological contracts with organization and take a sense of ownership, enhancing the affective commitment of employees (Ohana, 2015; Parzefall and Hakanen, 2010). Taken together, we hypothesize the following:

*H3*: Voice behavior mediates the relationship between digital leadership and the affective commitment of new generation employees.

### 2.5 The chain mediating role of employee empowerment and voice behavior

**Figure 1:**
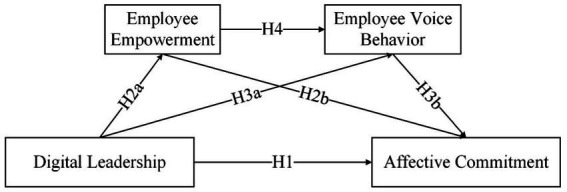
The theoretical model.

To complete our hypothesized model, we further predict that employee empowerment can positively influence the voice behavior of new generation employees. New generation employees with high perceptions of empowerment tend to feel supported and trusted by the organization. As a result, a high sense of security and efficacy motivates them to be more vocal. Moreover, employee empowerment creates a perception of owning power in the new generation of employees, further engaging employees in their work. Continuous work engagement also makes employees more aware of the deficiencies in their work processes and environment, which in turn leads them to think about how to improve the status quo of the organization (Figure 1).

Consistent with affective events theory, digital leaders trigger employee empowerment and employee voice behavior (affective events), which subsequently influences the affective commitment of new generation employees (affective responses). Specifically, digital leaders excel at empowerment in digital contexts, enhancing new generation employees’ sense of control and effectiveness at work. With access to more digital resources and information, employees dare to voice for the development of the organization, which in turn generates an insider identity and affective commitment. Based on this, we hypothesize the following:

*H4*: Employee empowerment and voice behavior serve as chain mediators between leaders’ digital leadership and the affective commitment of new generation employees.

## 3 Methods

### 3.1 Participants and procedure

Compared to the central and eastern regions, enterprises in Southwest China began their digitalization relatively late. However, in recent years, the potential for digital transformation in specialized industries has grown significantly with strong policy support and regional collaboration, involving traditional manufacturing, green agriculture, cultural tourism, energy, and other advantageous industries. A large number of digital talents are gathered here and the management of digital talents has made a difference. Thus, we select 12 leading enterprises from these characteristic industries of the southwest region. They prioritize the cost-effectiveness of technology in practical operations and the cultivation of digital talent. Then, this study mainly adopts a questionnaire survey to the employees in the 12 selected enterprises, who are intellectual workers with professional and technical skills or management skills. They work mainly in the form of online collaboration and interact with leaders more frequently.

At the beginning of the research, we communicate with the human resources department of the surveyed company, and after receiving permission for the research, number the employees of the company. Paper questionnaires are then distributed to the respondents according to the number, and the respondents are informed that the data are confidential and used only for academic research. To minimize the impact of common methodological bias issues, a two-time points data collection method with an interval of 10 days is used in this study. At time point 1, the survey is for employees, including basic socio-demographic information, digital leadership and voice behavior. At time point 2, the survey is administered to the employees, which included affective commitment and employee empowerment.

In the first survey, 840 employee questionnaires are distributed on-site, and 697 valid questionnaires are returned. In the second survey, targeted distribution is made to employees who provided valid questionnaires in the first survey, and a total of 593 valid questionnaires are returned. Excluding employees born before 1990, as well as invalid questionnaires that are abandoned or take less than 2 minutes to complete. Finally, 408 valid questionnaires are obtained, with a return rate of 48.571%. In terms of sample structure, female employees predominate, accounting for 54.902% of the total sample. In terms of education background, bachelor’s degree predominates, with 71.324% of the total sample size. In terms of working years, 3 ~ 5 years predominates, with 43.627% of the total sample. Besides, the majority of respondents work in privately owned enterprises, with 53.676% of the total sample. The size of companies in the sample is mainly between 300 ~ 1,000, with a rate of 39.706%.

### 3.2 Measurements

The measurement scales used in this study are mature or widely used by scholars both at home and abroad. Each item is scored on a Likert 5-point scale (1 = strongly disagree and 5 = strongly agree), measuring the main four variables: digital leadership, affective commitment, employee empowerment, and voice behavior.

#### 3.2.1 Digital leadership

The 6-dimension measurement scale with 18 items developed by Roman et al. (2019) is used, including the ability to digital communication, digital social, digital team, digital change, digital technology and digital trust. Combined with affective events theory and the online work contexts in Chinese enterprises, we have revised this scale. Representative items are “In online communication, my leader can orderly organize activities and timely receive feedback from employees”, “In online communication, my leader is able to motivate team members online to make it work effectively.” The Cronbach’s α is 0.912.

#### 3.2.2 Affective commitment

The measurement of digital leadership is based on the scale compiled by Tsui et al. (1997) which contains nine items. According to the characteristics of new generation employees, we have revised some items. The representative item is “I am willing to go above and beyond for the success of the organization.” The Cronbach’s α is 0.919.

#### 3.2.3 Employee empowerment

Four-dimension measurement scale with 16 items developed by Rogers and Singhal (2003) is used, including self-efficacy, influence, meaning of work, and autonomy. According to affective events theory and the relationships of concepts, we further revise this scale. The representative item is “I possess the authority to make decisions, even if they are not entirely correct.” The Cronbach’s α is 0.899.

#### 3.2.4 Employees voice behavior

The measurement of employee voice behavior is based on the scale developed by Liang et al. (2012) which contains ten items. Combined with affective events theory and characteristics of new generation employees, we have revised some items. Representative items are “I often timely discourage behaviors of other employees that negatively affect work efficiency within the organization,” “I often propose constructive suggestions to help the company achieve its goals.” The Cronbach’s α is 0.890.

#### 3.2.5 Control variables

This study selects gender, tenure, and education at the employee level, and size and ownership type at the enterprise level as control variables. It ensures that the relationship between the core variables is not obscured by other factors.

### 3.3 Reliability and validity test

In this research, SPSS 26.0 and AMOS 24.0 are used to conduct reliability and validity tests. The results are shown in Table 1. Cronbach’s α of four main variables are all above the recommended value of 0.7, which means that the scales have good reliability. In addition, the factor loading coefficients, composite reliability and average variance extracted are also higher than their standard values, indicating that the scales have good reliability and convergent validity. Besides, according to the result of Table 2, the square roots of the average variance extracted are all above their correlation coefficients, thus indicating a good discriminant validity in each scale.

**Table 1:** Measurement, factor analysis and reliability test (*N* = 408).

Variables	Cronbach’s α	Factor loading	Composite reliability	Average variance extracted
Digital leadership	0.912	0.604 ~ 0.775	0.949	0.554
Affective commitment	0.919	0.649 ~ 0.816	0.922	0.570
Employee empowerment	0.899	0.583 ~ 0.826	0.939	0.508
Employee voice behavior	0.890	0.562 ~ 0.822	0.922	0.545

**Table 2:** The descriptive statistics and correlation analysis.

Variable	1 Gender	2 Education	3 Time	4 Ownership	5 Size	6 DL	7 EE	8 EVB	9 AC
Mean	0.450	2.890	3.280	2.030	2.820	4.039	4.003	3.879	3.870
SD	0.498	0.584	0.911	0.908	0.835	0.531	0.499	0.623	0.712
Kurtosis	−1.971	1.644	−0.054	0.650	−0.811	−0.309	−0.439	0.088	0.892
Skewness	0.198	−0.650	−0.228	0.981	−0.086	−0.670	−0.538	−0.682	−1.047
1		−0.07	0.005	−0.067	0.029	−0.024	0.063	0.020	−0.143**
2			−0.061	0.077	0.029	0.019	0.051	0.042	0.009
3				−0.062	0.188**	0.054	0.115*	0.100*	0.103*
4					−0.219**	0.01	0.051	0.053	0.001
5						0.016	0.036	0.011	0.023
6						**(0.744)**	0.589**	0.616**	0.524**
7							**(0.713)**	0.671**	0.533**
8								**(0.738)**	0.640**
9									**(0.755)**

*N* = 408; DL, Digital Leadership; EE, Employee Empowerment; EVB, Employee Voice Behavior; AC, Affective Commitment; *p < 0.05; **p < 0.01; ***p < 0.001. Bold values means square roots of the average variance extracted are all above their correlation coefficients, it indicates a good discriminant validity in each scale.

### 3.4 Common method bias test and confirmatory factor analysis

Considering the potential impact of common method bias on the measurement model, this study conducts Harman’s single-factor test. An unrotated exploratory factor analysis is performed on all questionnaire items, extracting eight factors with eigenvalues greater than 1. The total variance explained is 65.452%, with the largest single factor accounting for 31.939% of the variance. This is below the threshold of 40% and less than 50% of the total variance explained, indicating that there is no significant common method bias.

Meanwhile, to assess the discriminant validity of the scales, this study employs confirmatory factor analysis (CFA) to test the model. As shown in Table 3, the four-factor model (*X*^2^/DF = 1.461, TLI = 0.939, CFI = 0.945, RMSEA = 0.034) achieves ideal fit indices and is significantly better than the other four alternative factor models, indicating that the four variables have good discriminant validity.

**Table 3:** Confirmatory factor analysis (CFA) results of measurement models.

Model	*X*^2^/DF	TLI	CFI	RMSEA
Model 1: DL + EE + EVB + AC	3.901	0.619	0.633	0.084
Model 2: DL, EE + EVB + AC	2.943	0.745	0.758	0.069
Model 3: DL, EE, EVB + AC	2.138	0.850	0.862	0.053
Model 4: DL, EE, EVB, AC	1.461	0.939	0.945	0.034

*N* = 408; DL, Digital Leadership; EE, Employee Empowerment; EVB, Employee Voice Behavior; AC, Affective Commitment; +: Two factors combined as one.

### 3.5 Descriptive analysis and correlation analysis

The means, standard deviations and correlation coefficients of the variables in this study are shown in Table 2. Digital leadership is positively correlated with employee empowerment ( γ =0.589, *p* < 0.01), voice behavior ( γ =0.616, *p* < 0.01) and affective commitment ( γ =0.524, *p* < 0.01). Employee empowerment is positively correlated with voice behavior ( γ =0.671, *p* < 0.01) and affective commitment ( γ =0.533, *p* < 0.01). Voice behavior is also positively correlated with affective commitment ( γ =0.640, *p* < 0.01). The results of the above analysis lay a robust foundation for the subsequent hypothesis testing.

## 4 Results

In this research, the full hypothesized model is tested using SPSS 26.0 and AMOS 24.0. This study employs regression analysis and Bootstrapping (repeated sampling 10,000 times) to test the model. In traditional regression analysis, hypothesis testing often relies on the hypothetical distribution of parameters, so a large sample size is required to ensure accurate results. If the sample size is small or there is a nonlinear relationship, bootstrapping can verify the complex model by multiple resampling, which improves the reliability of regression analysis results. In Tables 4, 5, after controlling for variables such as gender and education, digital leadership has a significant positive impact on the affective commitment of the new generation employees ( β = 0.517, *p* < 0.001), confirming Hypothesis 1 (Model 4).

**Table 4:** Results of variable regression analysis.

Variable	EE	EVB	AC				
	Model 1	Model 2	Model 3	Model 4	Model 5	Model 6	Model 7
DL	0.582***	0.612***	0.338***	0.517***	0.434***	0.200***	0.167***
EE			0.470***		0.474***		0.115**
EVB						0.519***	0.463***
Gender	−0.044	0.040	0.061	−0.132**	−0.118**	−0.153***	−0.146***
Education	0.038	0.034	0.016	−0.005	−0.018	−0.023	−0.025
Time	0.086**	0.073	0.033	0.075	0.046	0.037	0.031
ownership type	0.049	0.050	0.027	−0.007	−0.023	−0.033	−0.036
Size	0.022	−0.004	−0.014	0.003	−0.004	0.005	0.002
R^2^	0.361	0.389	0.530	0.298	0.368	0.462	0.469
F	37.691***	42.517***	64.503***	28.341***	33.326***	49.160***	44.037***

*N* = 408; DL, Digital Leadership; EE, Employee Empowerment; EVB, Employee Voice Behavior; AC, Affective Commitment; Bootstrap = 10,000 times. ***p < 0.001; **p < 0.01; *p < 0.05.

**Table 5:** The mediating effect of employee empowerment and voice behavior.

Model path	β	Boot SE	95% Confidence interval		Percentage
			Lower	Upper	
DL → AC	0.225	0.065	0.096	0.353	32.37%
DL → EE → AC	0.090	0.046	0.001	0.180	12.95%
DL → EVB → AC	0.210	0.047	0.126	0.308	30.22%
DL → EE → EVB → AC	0.170	0.035	0.106	0.244	24.46%

*N = 408; DL, Digital Leadership; EE, Employee Empowerment; EVB, Employee Voice Behavior; AC, Affective Commitment; Bootstrap = 10,000 times.*

Drawing on Baron’s approach to the mediating effect test, this paper evaluates the mediating role of employee empowerment through the hierarchical regression method (Baron and Kenny, 1986). First, in Model 1 of Table 4, digital leadership had a significant positive effect on employee empowerment ( β = 0.582, *p* < 0.001); Second, according to Model 5 of Table 4, employee empowerment had a significant positive effect on affective commitment ( β = 0.474, *p* < 0.001). Finally, compared to Model 4, the impact of digital leadership on affective commitment decreases with the addition of mediating variables ( β =0.517→ β =0.434), which verifies the mediating role of employee empowerment. To further test the model, we select Model 4 of the Macro Process 3.3 developed by Hayes for 10,000 replicate sampling with 95% confidence intervals. If the confidence interval does not contain 0, we consider the model is effective. According to Table 5, the mediating effect value of employee empowerment between digital leadership and affective commitment is 0.090, with a 95% confidence interval of [0.001, 0.180], not containing 0. It indicates that the mediation effect is significant, thus confirming Hypothesis 2.

The mediating effect of voice behavior is also examined. As indicated by Models 2 and 6 in Table 4, digital leadership has a significant positive impact on employee voice ( β = 0.612, *p* < 0.001); voice behavior has a significant positive effect on affective commitment ( β = 0.519, *p* < 0.001). Similarly, in Model 6, we can see the coefficient of digital leadership influencing affective commitment is 0.200, less than 0.517 in Model 4, which validates the mediating effect of voice behavior. Again, we use the Bootstrapping method developed by Hayes. As shown in Table 5, the mediating effect value of employee voice between digital leadership and affective commitment is 0.210, with a 95% confidence interval of [0.126, 0.308], not containing 0, thus confirming the mediating effect of voice behavior. Hypothesis 3 is verified.

Consequently, the chain mediating effect of employee empowerment and employee voice behavior is tested. As shown in Model 3 of Table 4, employee empowerment has a significant positive effect on voice behavior ( β = 0.470, *p* < 0.001); as shown in Model 7, when digital leadership, employee empowerment, and voice behavior are taken as independent variables, their effects on affective commitment remains significant ( β _1_ = 0.167, *p* < 0.001; β _2_ = 0.115, *p* < 0.005; β _3_ = 0.463, *p* < 0.001). For more complex chain mediating models, it is more necessary to verify the regression model through bootstrapping. We select Model 6 of the Macro Process 3.3 for 10,000 replicate sampling with 95% confidence intervals. As is shown in Table 5, the chain mediating effect value of employee empowerment and voice behavior between digital leadership and affective commitment is 0.170, with a 95% confidence interval of [0.106, 0.244], not containing 0, thus confirming Hypothesis 4.

## 5 Discussion

In this study, digital leadership, employee empowerment, employee voice behavior, and affective commitment of new generation employees are included in the same research framework, and the hypotheses are verified by empirical analysis with the 408 valid data. First, digital leadership has a significant positive impact on the affective commitment of new generation employees. That is, as a key element of major events in the workplace, digital leadership can trigger events that align with the preferences of new generation employees and inspire their positive emotions. Reviewed with the traits of new generation employees, digital leadership is able to demonstrate a higher level of affective commitment by meeting their needs of self-actualization, respect, and autonomy. Second, employee empowerment and voice behavior partially mediate the relationship between digital leadership and affective commitment. It seems to follow that if digital leaders provide online learning platforms, are open to innovative ideas and emphasize personalized feedback and incentives, new generation employees will feel a sense of self-efficacy and psychological safety, which promote the sense of empowerment and voice efficacy. These perceptions will drive employees to act as voice behaviors, which further generates interactions with digital leaders and meets their needs for self-realization, and self-improvement and being respected. Eventually, their affective commitment is enhanced. Third, employee empowerment and voice behavior serve as the chain mediating role in the relationship between digital leadership and the affective commitment of new generation employees. It further reveals the deeper interactive relationships and provides theoretical support for understanding these relationships.

### 5.1 Theoretical implications

First, the findings demonstrate the impact of digital leadership on the affective commitment of new generation employees. It not only enriches the theoretical explanation of the drivers of affective commitment among new generation employees but also opens up a space for exploring the impact of digital leadership. Based on that, we construct the relationship between digital leadership and the new generation employees and extend the online interactions contexts of affective events theory. Besides, our study supplements the impact of digital leaders and affective connections with employees. Meanwhile, we reveal the characteristics of new generation employees and their affective commitment, which adds to the research on the emotional motivations of behaviors among the new generation employees and builds connections related to affective events with leaders. It further lays a theoretical foundation for future research on their workplace behaviors.

Second, we introduce employee empowerment as an internal mechanism to explain the impact of digital leadership on the affective commitment of new generation employees. According to affective commitment, digital leadership will drive something to happen that inspires emotional responses from employees. That is, employee empowerment is an interactive event initiated by the leader, which is aligned with the goals and values of new generation employees and further triggers a positive emotional experience for them. These results expand the study of emotional responses of empowerment among new generation employees, which paves the way for future research on the motivation of them. Additionally, this paper examines the mediating pathways of employee voice behavior. Traditional employees are likely to advise with a high perception of affective commitment (Machokoto, 2019; Zhou et al., 2021). While for new generation employees, only based on sufficient interactions, especially highly involved voice behavior, can the new generation employees form affective commitment. Combined with the traits of new generation employees, we focus on the antecedent of affective commitment among new generation employees, which is largely overlooked in comparison to that of traditional employees. We supplement the characteristics and antecedent of affective commitment of new generation employees, which provides a theoretical foundation for the study of motivation and retention among new generation employees. Moreover, it widens the scope of affective events theory toward the group of new generation employees and certifies that interactive affective events can engender their affections in similar pathway.

Further, we confirm the chain mediating roles of employee empowerment and voice behavior. It reflects that the interactive relationship between leaders and employees can be deepened by behavior. On the one hand, employee voice behavior can strengthen the emotional connections between digital leaders and new generation employees. Especially for new generation employees with unique traits, they will accept more leaders’ behavioral events and respond to them. A logical chain of “leaders’ behavioral events—employees’ behavioral responses to suggestions—employees’ emotional responses” is built by the affective events theory in digital contexts. This reinforces the theory in the event-emotion interactions between leaders and employees. On the other hand, employee empowerment reflects the perceptions from the interactive events with leaders, which echoes a logical chain of “leaders’ behavioral events— employees’ recognition— employees’ affections.” It strengthens the roles of personal traits and recognitions between events and emotions.

### 5.2 Practical implications

To sum up, this study provides some practical implications.

First, we discover that digital leaders can increase the affective commitment of new generation employees. Therefore, leaders must actively promote their digital leadership as well as take notice of the different traits and emotional responses of new generation employees. Employees’ autonomy and self-realization should be respected and valued. At the same time, digital and personalized measures should be taken by leaders to motivate and communicate with new generation employees.

Second, the mediation roles of employee empowerment and voice behavior are revealed and provide some constructive implications. For leaders, it is vital to empower employees and focus on their growth and development. If leaders support a fair and transparent empowerment process, employees should be more likely to interact with leaders in ways that are perceived as respected and motivated. Besides, Leaders should also create multiple channels for employees to two-way communications, establish personalized incentives and policies, and allow employees to participate in organizational management. It is essential for organizations and leaders to understand what triggers new generation employees to behave in a way that commits to their organizations. For new generation employees, they should observe their unique needs such as being respected and self-realization, and then try their best to participate in the management, especially advice. Meanwhile, they should have a bit more patience for the adaption of organizations and leaders. It goes through a period that employees experience interactive events with leaders and have certain cognitions and emotions.

Third, we further reveal the chain mediating role of employee empowerment and voice behavior in the relationship between digital leadership and the affective commitment of new generation employees. Namely, new generation employees with a higher perception of empowerment are likely to take the voice behaviors for leaders. New generation employees should be open to the leaders’ power and embrace the knowledge and skills through digital resources to increase the perceptions of empowerment and potency.

### 5.3 Limitations and directions for future research

This study inevitably has certain limitations that warrant further exploration and improvement. First, the study uses cross-sectional survey data, but the impact of digital leadership on affective commitment may have a temporal lag, which is likely to affect the statistically significant results. Thus, future research can adopt longitudinal studies to examine the relationships between variables over time. Second, given that external variables, such as emotional intelligence and power distance, may influence the relationships of main variables. Future studies will be intriguing if related boundary factors are involved.

## Data Availability

The original contributions presented in the study are included in the article/supplementary material, further inquiries can be directed to the corresponding author.
